# The p53/ZEB1-PLD3 feedback loop regulates cell proliferation in breast cancer

**DOI:** 10.1038/s41419-023-06271-4

**Published:** 2023-11-17

**Authors:** Bo-Wen Liu, Ning Sun, Hui Lin, Xue-Jie Zhou, Hai-Yan Ma, Xin Wang, Xu-Chen Cao, Yue Yu

**Affiliations:** 1 The First Department of Breast Cancer, Tianjin Medical University Cancer Institute and Hospital, National Clinical Research Center for Cancer, Tianjin, 300060 China; 2 Key Laboratory of Breast Cancer Prevention and Therapy, Tianjin Medical University, Ministry of Education, Tianjin, 300060 China; 3 Key Laboratory of Cancer Prevention and Therapy, Tianjin, 300060 China; 4 Tianjin’s Clinical Research Center for Cancer, Tianjin, 300060 China; 5 Department of Thyroid and Breast Surgery, The Second Hospital of Tianjin Medical University, Tianjin, 300211 China; 6 Department of Surgical Oncology, Taizhou Hospital of Zhejiang Province affiliated to Wenzhou Medical University, Wenzhou, Zhejiang 317099 China

**Keywords:** Breast cancer, Tumour-suppressor proteins, miRNAs, Mitosis

## Abstract

Breast cancer is the most prevalent cancer globally, endangering women’s physical and mental health. Phospholipase D3 (PLD3) belongs to the phosphodiesterase family (PLD). PLD3 is related to insulin-mediated phosphorylation of the AKT pathway, suggesting that it may play a role in the occurrence and development of malignant tumors. This study may further explore the molecular mechanism of PLD3 inhibiting breast cancer cell proliferation. In this study, we demonstrated that PLD3 and miR-6796 are co-expressed in breast cancer. PLD3 can bind with CDK1 and inhibit its expression, leading to mitotic arrest and inhibiting breast cancer proliferation. Wild-type p53 regulates PLD3 and miR-6796 expression by competitively binding to the PLD3 promoter with ZEB1. DNMT3B, as the target gene of miR-6796, is recruited into the PLD3 promoter by combining with ZEB1 to regulate the DNA methylation of the PLD3 promoter and ultimately affect PLD3 and miR-6796 expression. In conclusion, we revealed the role and molecular mechanism of PLD3 and its embedded miR-6796 in breast cancer proliferation, providing clues and a theoretical foundation for future research and development of therapeutic targets and prognostic markers for breast cancer.

## Introduction

Breast cancer is the most prevalent cancer in the world. Although systematic treatment can increase the five-year survival rate of breast cancer patients to 90%, patient prognosis with advanced metastasis is still poor, and recurrence and metastasis remain the leading causes of death in breast cancer patients [1]. Uncontrolled growth and proliferation are biological characteristics of malignant tumor cells and are one of the leading causes of treatment failure in patients. It is characterized by the up-regulation of cyclin-dependent kinase (CDK), increased DNA synthesis, and normal apoptosis pathway inhibition [2]. Our understanding of the molecular mechanisms of breast cancer occurrence and progression is still limited [3]. It is necessary to understand its molecular mechanism better, to explore and identify more specific and sensitive markers, to provide accurate treatment, and to improve the prognosis and survival rate of patients.

The Phospholipase D3 (PLD3) gene, also known as HU-K4, is located on human chromosome 19q13.2. The PLD family has six members, PLD1 to 6, each with two H(X)K(X4)D (HKD) domains [4]. PLD2 activity increases in various tumors, including breast cancer, colon cancer, gastric cancer, and renal cell carcinoma [5]. The increased PLD2 activity indicates breast cancer cells resist the traditional mTOR inhibitor rapamycin [6]. PLD1 can activate PLD2 activity by increasing phosphatidylinositol (PIP2) levels [7]. Despite having a similar structure to PLD2, PLD3 lacks hydrolase activity, implying that phosphatidic acid (PA) cannot be produced by hydrolyzing phosphatidylcholine [8]. PLD3 protein is highly expressed in the central nervous system, smooth muscle, skeletal muscle, cardiac muscle, lung tissue, epididymis, and other tissues. PLD3 research is currently focused on the regenerative potential of hematopoietic stem cells, actin production in myocytes [9], and Alzheimer’s disease [10], with no reports of tumor-related studies.

MicroRNA (miRNA) is a single-stranded small non-coding RNA with an average length of 21 to 23 bp. Although microRNA does not code for proteins, it can regulate the stability of target genes by binding to complementary seed sequences [11, 12]. Dozens of breast cancer-related miRNAs regulate breast cancer cell proliferation, invasion, metastasis, and other biological behaviors [13, 14]. miRNAs are classified as intergenic or intronic based on their coding positions [15]. Intronic miRNAs that depend on the transcription of encoded genes typically co-express with their host genes to perform specific physiological or pathological functions. For example, miR-208 and its host gene MYH6 are involved in the occurrence of myocardial hypertrophy and fibrosis [16]. miR-33 and its host gene SREBF1 co-regulate cholesterol metabolism [17]. miR-126 assists its host gene EGFL7 in inhibiting prostate cancer invasion and metastasis [18]. miR-6796 was discovered in exons 6 and 7 of the PLD3 gene after analyzing its genome structure. Few studies have been reported the function of miR-6976, while a recent study demonstrated that miR-6976-5p could be sponged by circSKA3 and associated with outcomes of ischemic stroke by regulating matrix metalloproteinase 9 expression [19]. However, there is no available information regarding the role of miR-6796 in breast cancer tumorigenesis and development.

This study aims to reveal the role and molecular mechanism of PLD3 and its embedded miR-6796 in breast cancer proliferation, thereby providing clues and a theoretical foundation for future research and development of therapeutic targets and prognostic markers.

## Results

### PLD3 functioned as a tumor suppressor gene in breast cancer

**Fig. 1:**
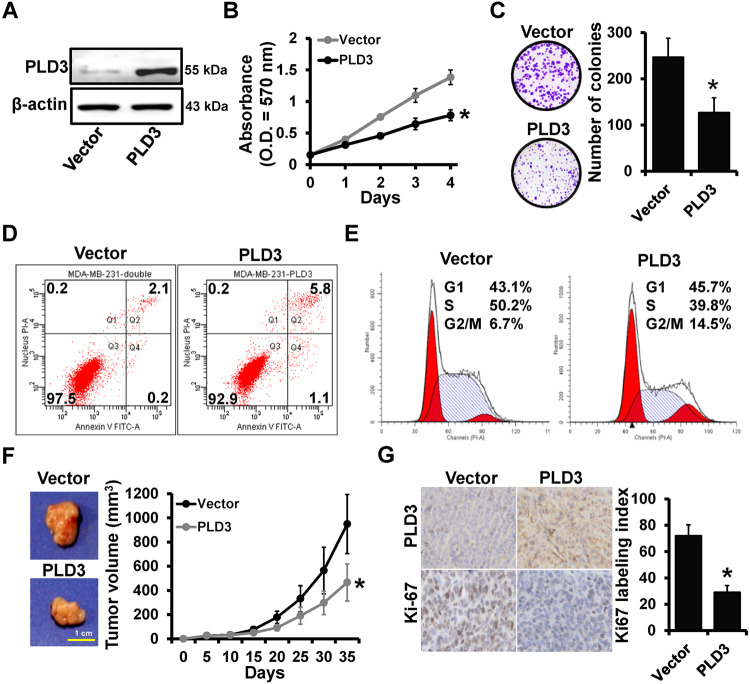
PLD3 functioned as a tumor suppressor gene in breast cancer. **A** PLD3 expression in stable PLD3-overexpression cells (231-PLD3) and control cells (231-Vector) was determined by the western blot. MTT (**B**) and colony-formation (**C**) analyzed the proliferation of the cells described in (**A**). Flow cytometry analyzed the apoptosis (**D**) and the cell cycle distribution (**E**) of the cells described in (**A**). **F** Tumor growth curves of the subcutaneous tumor made of the cells described in (**A**) at the indicated times and dissected tumors photographed at the harvest time, each group included 6 mice. **G** IHC staining analyzed the expression level of Ki-67 in breast cancer tissues with high or low PLD3 expression, the representative IHC image of each mouse has been selected for statistical analysis. *P < 0.05.

MDA-MB-231 was used to generate stable PLD3-overexpression cells (231-PLD3) and control cells (231-Vector) via lentiviral infection (Fig. 1A) to explore the potential role of PLD3 in breast cancer progression. Proliferation assays indicated that PLD3 overexpression could inhibit MDA-MB-231 cell viability and colony formation ability in vitro using MTT (Fig. 1B) and colony formation assays (Fig. 1C). Furthermore, PLD3 overexpression increased the proportion of apoptotic cells (Fig. 1D) and caused cell cycle arrest at the G2/M phase (Fig. 1E). These results indicate that PLD3 induces cell cycle arrest and cell apoptosis in breast cancer. The 231-PLD3 and 231-Vector cells were injected into mice mammary fat pads to evaluate the effect of PLD3 expression on tumor growth in vivo. Tumor growth was measured 35 days after injection. As hypothesized, we observed that orthotopic tumor volume was significantly lower in the PLD3-overexpression group than in the control group (Fig. 1F). IHC staining revealed that Ki-67 was down-regulated in tumors from 231-PLD3 mice compared to 231-Vector mice (Fig. 1G).

Afterward, we investigated whether PLD3 depletion could influence breast cancer progression. The siRNAs targeting PLD3 were transfected into MCF-7 cells (Fig. S1). MTT (Fig. S1B) and colony formation (Fig. S1C) assays indicated that PLD3 depletion significantly promoted breast cancer cell proliferation. Furthermore, flow cytometry analysis revealed that PLD3 depletion decreased the proportion of G2/M phase cells in PLD3-depleted cells compared to control cells (Fig. S1D). These results indicate that PLD3 is a tumor suppressor gene in breast cancer.

### PDL3 overexpression induces mitotic arrest in breast cancer

**Fig. 2:**
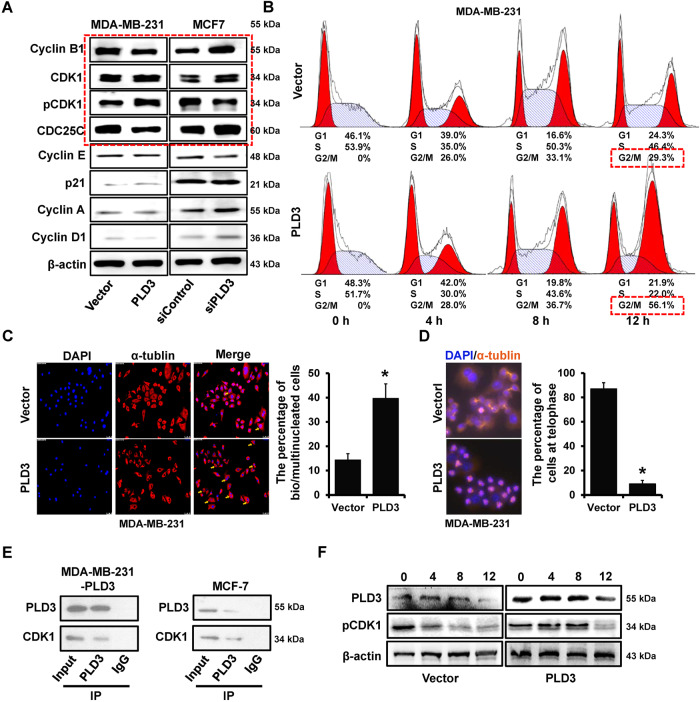
PDL3 overexpression induces mitotic arrest in breast cancer. **A** Cell cycle checkpoint related proteins expression level in PLD3-overexpression cells and PLD3‑depleted cells, as determined by western blot. **B**–**D** The double thymidine cell cycle synchronization method or thymidine nocodazole cell cycle synchronization method was used to synchronize breast cancer cells in G1/strand or pre-mitotic metaphase. The effect of PLD3 expression levels on breast cancer cell cycle distribution was evaluated using flow cytometry (**B**). The effect of PLD3 on multinucleation was evaluated by IF (**C**). The PLD3 effect on mitosis was evaluated using IF (**D**). **E** PLD3 bound to CDK1 in PLD3 overexpression cells, as determined using IP. **F** The pCDK1 protein expression levels in 231-PLD3 and 231-Vector cells at 0, 4, 8, and 12 h after the release of synchronized breast cancer cells in G1/strand, as determined using western blot. *P < 0.05.

We detected the cell cycle-related protein expression levels after PLD3 overexpression or knockdown to verify the PLD3 effect on the cell cycle. The western blot demonstrated that PLD3 increased CDK1 protein phosphorylation and lowered the cyclin B1 production, which are the detection points at the G2/M phase of cell cycle (Fig. 2A). The double thymidine cell cycle synchronization or thymidine nocodazole cell cycle synchronization methods were used to synchronize breast cancer cells in G1/strand or pre-mitotic metaphase. The cell cycle distribution was determined at 0, 4, 8, and 12 h following release. In the G2/M phase, the cell proportions in the 231-PLD3 group were considerably higher than in the 231-Vector group (56.1% vs. 29.3%, at the12 h after release, Fig. 2B).

Furthermore, the multinucleation cell proportion was also increased in the 231-PLD3 group compared with the 231-Vector group (Fig. 2C). Besides, after 0.5 h of release, IF detection revealed that most of the cells in the 231-Vector group were at the end of mitosis, whereas the cells in the 231-PLD3 group were still in the middle of mitosis (Fig. 2D). The protein spectrum data analysis revealed that PLD3 might bind to CDK1, and IP in PLD3-overexpressed MDA-MB-231 and MCF7 cell lines further verified this association (Fig. 2E). In addition, the western blot demonstrated that PLD3 overexpression up-regulated pCDK1 expression, which led to CDK1 activity reduction in MDA-MB-231 (Fig. 2F). The above results demonstrated that PLD3 overexpression could inhibit breast cancer cell proliferation by decreasing CDK1 activity, leading to mitotic arrest.

### Wild-type p53 transactivates PLD3 expression

**Fig. 3:**
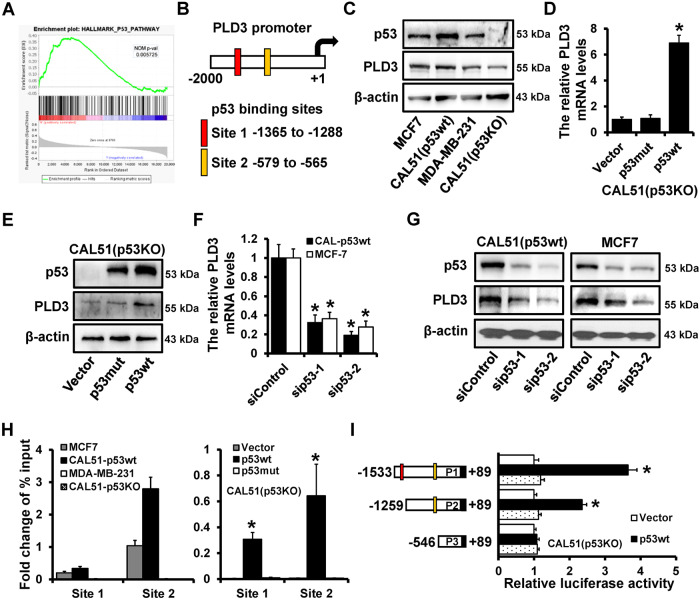
Wild-type p53 transactivates PLD3 expression. **A** PLD3 expression is correlated with the activity of the p53 signaling pathway using GSEA. **B** Two potential p53‑binding sites located in the PLD3 promoter region. **C** p53 and PLD3 protein expression in normal breast cell line MCF10A and breast cancer lines as determined using western blot. The effects of wild-type and mutant p53 on PLD3 expression were determined using RT-qPCR (**D**) and western blot (**E**). The effects of knockdown p53 on PLD3 expression levels were determined using RT-qPCR (**F**) and western blot (**G**). **H** The interaction between p53 and PLD3 promoter region in indicated cells was verified using ChIP analysis. **I** Dual-luciferase reporter assays were used to analyze the regulation of PLD3 promoter activity by p53wt. Several luciferase reporter plasmids containing different deletions in the PLD3 promoter region were co-transfected with a p53wt-expressing plasmid into CAL51 (p53KO) cells. *P < 0.05.

The Gene Set Enrichment Analysis (GSEA) of the PLD3 differentially expressed genes revealed enrichments in the p53 signaling pathway in breast cancer samples with high PLD3 expression, allowing us to examine further the molecular mechanism of PLD3 that limits breast cancer cell proliferation (Fig. 3A). Further analysis by JASPAR website (https://jaspar.genereg.net/) of the PLD3 promoter sequence (−2000 ~ +1 to TSS) revealed two potential p53 binding sites (Fig. 3B). Western blot was used to detect PLD3 expression in breast cancer cells with varying p53 statuses. PLD3 has a high expression level in the p53 wild-type breast cancer cell lines MCF7, and CAL51 (p53wt) and a low expression level in the p53 mutant MDA-MB-231 and p53 deleted CAL51 (p53KO) breast cancer cell lines (Fig. 3C), indicating that p53 may be responsible for regulating PLD3 expression. PLD3’s mRNA and protein expression were dramatically up-regulated by overexpressing wild-type p53 (p53wt) in p53-deleted CAL51 (p53KO) cells, while mutant p53 (p53mut) had no discernible impact on PLD3’s expression (Fig. 3D, E). PLD3’s mRNA and protein expression levels were markedly reduced after p53 was knocked down in the breast cancer cell lines MCF7 and CAL51 (p53wt) (Fig. 3F, G). It was demonstrated that p53wt could regulate PLD3 expression.

Moreover, ChIP demonstrated that p53 could bind to the PLD3 promoter region in the p53wt breast cancer cells but not in p53mut breast cancer cells (Fig. 3H). We cloned several deletion mutants of the region starting 1533 bp upstream of the PLD3 promoter into the pGL3-basic reporter and transfected the reporters into CAL51 (p53KO) cells to reveal that p53wt regulates PLD3 expression transcriptionally. We performed luciferase assays to measure promoter activity. The p53wt overexpression significantly increased the luciferase activity of the constructs P1 and P2 but not that of the construct P3 (Fig. 3I). These results confirm that wild-type p53 can activate PLD3 expression via transcription.

### PLD3 promoter methylation regulates the expression of PLD3 and miR-6796

**Fig. 4:**
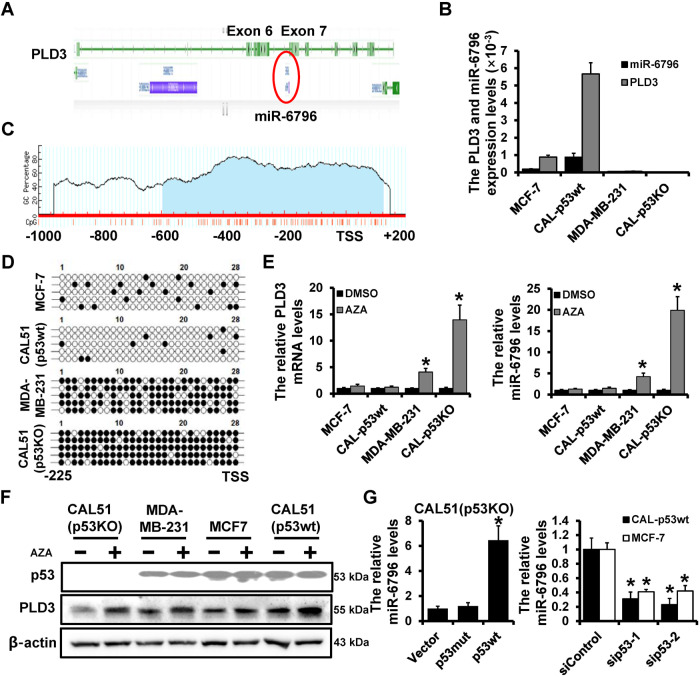
PLD3 promoter methylation regulates the expression of PLD3 and miR-6796. **A** Schematic diagram of the genomic structure of PLD3 and its chimeric miR-6796. **B** RT-qPCR analyzed PLD3 and miR-6796 mRNA expression in indicated cells. **C** CpG islands exist in the PLD3 promoter region. **D** BSP analyzed DNA methylation levels of PLD3 promoter in indicated cells. **E** RT-qPCR analyzed PLD3 mRNA and miR-6796 expression after AZA treatment in indicated cells. **F** PLD3 protein expression after AZA treatment in indicated cells, as determined using western blot. **G** RT-qPCR analyzed miR-6796 expression levels in overexpressing p53wt or p53mut CAL51 (p53KO) cells and p53-depleted MCF-7cells or CAL51 (p53wt) cells. *P < 0.05.

Intronic miR-6796 was discovered in exons 6 and 7 of the PLD3 gene after analyzing its structure (Fig. 4A). The PLD3 mRNA and miR-6796 expression levels were determined using RT-qPCR in breast cancer cell lines with varying p53 status, proving that PLD3 co-expressed with miR-6796. PLD3 mRNA and miR-6796 were substantially expressed in the p53 wild-type cells but not in the p53 mutant cells (Fig. 4B). The analysis performed by Meth Primer website (http://www.urogene.org/methprimer) demonstrated that CpG islands are present in the PLD3 promoter region (Fig. 4C), suggesting that PLD3 promoter DNA methylation regulates PLD3 and its embedded miR-6796. Furthermore, bisulfite sequence analysis of breast cancer cell lines revealed that the PLD3 promoter DNA was hypomethylated in p53wt cells with high PLD3 and miR-6796 expression. However, p53mut cells with low PLD3 and miR-6796 expressions were hypermethylated (Fig. 4D). The RT-qPCR results indicated that PLD3 mRNA and miR-6796 expression in p53mut cells was dramatically up-regulated after treatment with the methyltransferase inhibitor AZA, whereas the expression in p53wt cells did not change appreciably (Fig. 4E). Western blot analysis revealed that AZA increased PLD3 protein expression in p53wt cells (Fig. 4F). As already mentioned, wild-type p53 can activate PLD3 expression. RT-qPCR revealed that p53-deleted CAL51 (p53KO) cells, p53wt, could dramatically increase the miR-6796 expression level, whereas p53mut had no impact. In the p53wt breast cancer cell lines, p53 knockdown caused miR-6796 levels to be significantly down-regulated (Fig. 4G).

PLD3, as the host gene of miR-6796, acts as a tumor suppressor gene in breast cancer, but the role of miR-6796 in breast cancer has not been reported. MDA-MB-231 cells were transfected with miR-6796 expressing plasmid to generate stable miR-6796-overexpression cells (231-miR-6796) and control cells (231-Control) were generated via lentiviral infection. MCF7 cells were transfected with miR-6796 inhibitor to generate miR-6796-depleted cells (MCF7-anti-6796) and control cells (MCF7-Control) via transient transfection (Fig. S2A). Proliferation assays indicated that miR-6796 could inhibit cell viability (Fig. S2B) and colony formation (Fig. S2C) ability in vitro.

Furthermore, flow cytometric analysis indicated that miR-6796 induced cell cycle arrest at the G2/M phase in breast cancer (Fig. S2D, E). Immunofluorescence (IF) detection proved that miR-6796 increased the proportion of cell multinucleation (Fig. S2F). In vivo, tumor volume was significantly decreased in the miR-6796-overexpression group compared to the control group (Fig. S2G). These results suggest that PLD3 and its embedded miR-6796 are co-expressed and that the DNA methylation level of the PLD3 promoter regulates its expression level.

### ZEB1 recruits DNMT3B on the PLD3 promoter and represses its activity

**Fig. 5:**
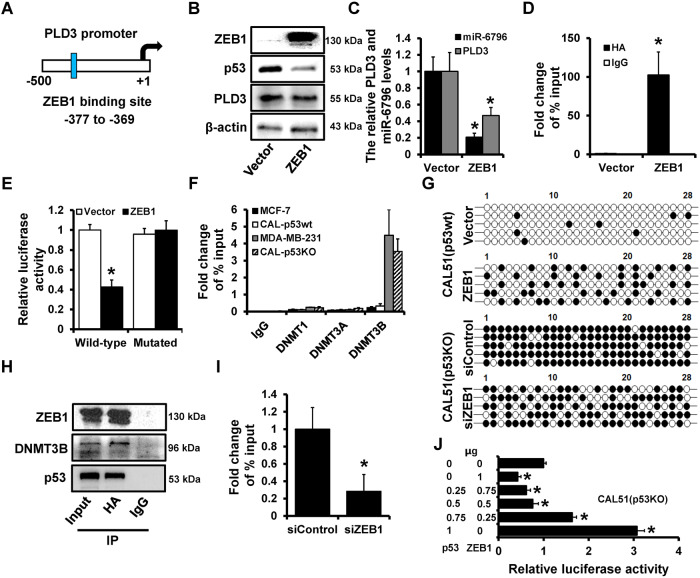
ZEB1 recruits DNMT3B on the PLD3 promoter and represses its activity. **A** Potential ZEB1‑binding sites located in the PLD3 promoter region. **B** The p53 and PLD3 protein expression levels in ZEB1-overexpression CAL51 (p53wt) cells and control cells were determined using western blot. **C** RT-qPCR analyzed PLD3 mRNA and miR-6796 the expression levels in ZEB1-overexpression CAL51 (p53wt) cells and control cells. **D** The interaction between ZEB1 and PLD3 promoter region in ZEB1-overexpression CAL51 (p53wt) cells and control cells verified using ChIP analysis. **E** Dual-luciferase reporter assays were used to analyze the regulation of PLD3 promoter activity by ZEB1. The wild-type predicted ZEB1 binding site was fused upstream of the Luc gene, and the construct with the ZEB1 binding site mutated was also created. The constructs were transfected into CAL51 (p53wt) cells with or without ZEB1 overexpression, and the luciferase activity was measured after 48 h. **F** The interaction between DNMTs and PLD3 promoter region in indicated cells, as verified using ChIP analysis. **G** BSP analyzed DNA methylation levels of PLD3 promoter in ZEB1-overexpression-CAL51 (p53wt) cells or ZEB1-depleted-CAL51 (p53KO) cells, as well as control cells. **H** ZEB1 bound to DNMT3B or p53 in ZEB1-overexpression-CAL51 (p53KO) cells determined using IP. **I** The interaction between DNMT3B and PLD3 promoter region in ZEB1-depleted-CAL51 (p53KO) cells, as verified using ChIP analysis. **J** Dual-luciferase reporter assays were used to analyze the regulation of PLD3 promoter activity by ZEB1 and p53. *P < 0.05.

Our previous studies indicated that ZEB1 could repress multiple miRNAs in breast cancer [20]. In this study, we sought to determine whether ZEB1 regulates PLD3 and miR-6796. JASPAR analyses revealed a potential ZEB1 binding site on the PLD3 promoter region (Fig. 5A). Previous studies have demonstrated that ZEB1 could modulate the DNA methylation level of the target gene promoter. Therefore, we hypothesized that ZEB1 could regulate the DNA methylation of the PLD3 promoter. Stable ZEB1-overexpression cells (ZEB1) and control cells (Vector) were generated using CAL51 (p53wt) through lentiviral infection. According to western blot analysis, ZEB1 overexpression reduced p53 and PLD3 expression (Fig. 5B). RT-qPCR analysis revealed that ZEB1 overexpression dramatically reduced the PLD3 mRNA and miR-6796 expression (Fig. 5C). ChIP revealed that ZEB1 could bind to the PLD3 promoter region in ZEB1-overexpressed CAL51 (p53wt) cells (Fig. 5D). Additionally, dual-luciferase reporter assays were used to analyze the regulation of PLD3 promoter activity by ZEB1. The constructs were transfected into CAL51 (p53wt) cells with or without ZEB1 overexpression, and the luciferase activity was measured after 48 h. The luciferase activity of the PLD3 promoter in ZEB1-overexpression cells was significantly inhibited (Fig. 5E). This study further examined how different DNMTs bind to the PLD3 promoter DNA. In p53mut cells with low PLD3 expression, only DNMT3B could bind to the PLD3 promoter DNA, detected by ChIP (Fig. 5F). According to the BSP results, ZEB1 overexpression could enhance DNA methylation of the PLD3 promoter, whereas ZEB1 knockdown could block promoter DNA methylation (Fig. 5G). In CAL51 (p53KO) cells with ZEB1 overexpression, the results of IP revealed that ZEB1 could bind with both DNMT3B and p53 (Fig. 5H). While down regulation of ZEB1 inhibited the capacity of DNMT3B on binding with PLD3 promoter, detected by ChIP analysis (Fig. 5I). According to these findings, ZEB1 could bind to DNMT3B, recruit it to the DNA region of the PLD3 promoter, and resulted in the PLD3 promoter hypermethylation and down-regulation.

### DNMT3B-mediated promoter hypermethylation represses PLD3 transcriptional activity

**Fig. 6:**
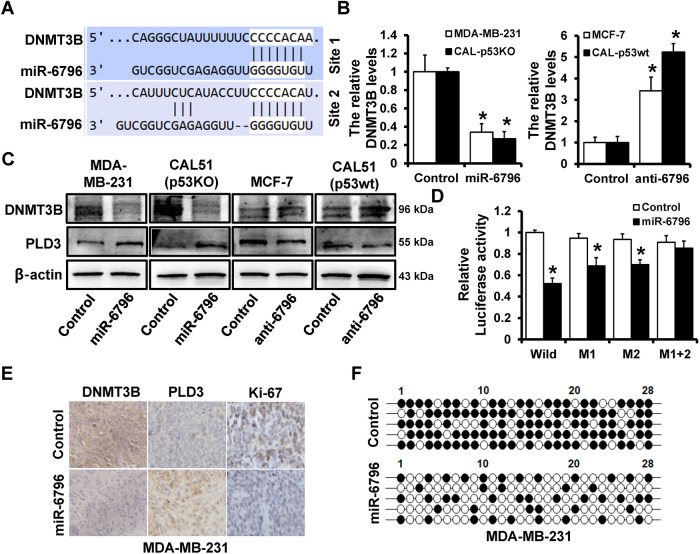
DNMT3B-mediated promoter hypermethylation represses PLD3 transcriptional activity. **A** Two miR-6796 binding sites located in DNMT3B 3’-UTR region. **B** RT-qPCR analyzed DNMT3B mRNA expression levels in miR-6796-overexpression cells or miR-6796‑depleted and control cells. **C** The DNMT3B or PLD3 protein expression levels in miR-6796-overexpression cells or miR-6796‑depleted, as well as control cells, as determined using western blot. **D** Dual-luciferase reporter analysis was performed to validate DNMT3B as a miR-6796 target. The wild-type 3’-UTR fragment containing the predicted miR-6796 target site of DNMT3B was fused downstream of the Luc gene, and constructs with the miR-6796 binding site of DNMT3B mutated were also created. The constructs were transfected into CAL51 (p53KO) cells with or without miR-6796 mimics, and the luciferase activity was measured after 48 h. **E** IHC staining analysis expression level of DNMT3B, PLD3, Ki-67 in breast cancer tissues with high or low miR-6796 expression. **F** BSP analyzed DNA methylation levels of PLD3 promoter in miR-6796-overexpression-MDA-MB-231 cells and control cells. *P < 0.05.

Two potential binding sites of miR-6796 on DNMT3B 3 ‘-UTR were predicted by Target Scan (https://www.targetscan.org/vert_72/) (Fig. 6A). Furthermore, RT-qPCR (Fig. 6B) and western blot (Fig. 6C) indicated that DNMT3B expression was decreased in miR-6796-overexpressed MDA-MB-231 and CAL51 (p53KO) cells but increased in miR-6796-depleted MCF7 and CAL51 (p53wt) cells compared to control cells. Wild-type or the miR-6796 binding site mutated DNMT3B 3’-UTR was cloned into the psiCHEK2 luciferase reporter to confirm this regulation further. Double luciferase assay revealed that miR-6796 overexpression could significantly inhibit the activity of DNMT3B 3’-UTR luciferase reporter plasmid, however, this effect was abolished when two sites mutated DNMT3B 3 ‘-UTR, in which the binding site for miR-6796 was inactivated by site-directed mutagenesis (Fig. 6D). IHC staining revealed that tumors from 231-miR-6796 mice had up-regulated PLD3 while down-regulated Ki-67 and DNMT3B compared to tumors from 231-Control mice (Fig. 6E). The BSP results demonstrated that the 231-miR-6796 group had lower levels of DNA methylation at the PLD3 promoter than the 231-Control group (Fig. 6F). These results indicate that DNMT3B is a target gene of miR-6796 and could mediate hypermethylation of the PLD3 promoter region, thereby inhibiting PLD3 transcriptional activity.

### PLD3 expression is low in breast cancer and associated with poor prognosis

**Fig. 7:**
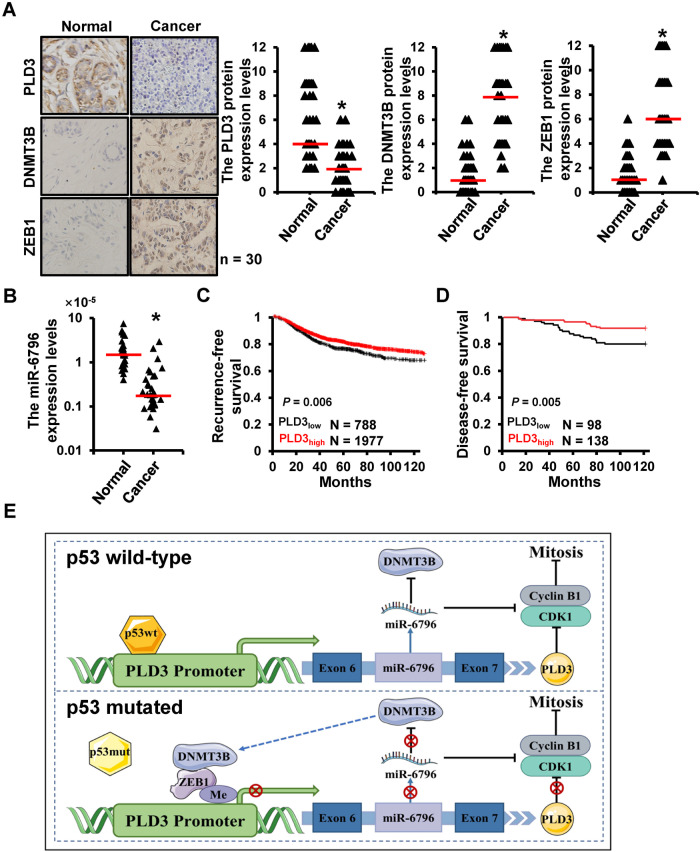
PLD3 was lowly expressed in breast cancer and associated with poor prognosis. **A** PLD3, DNMT3B, and ZEB1 expression levels were determined using IHC and RT-qPCR in primary breast cancer and the paired normal breast tissues (*N* = 30). **B** RT-qPCR analyzed miR-6796 expression levels in primary breast cancer and the paired normal breast tissues (*N* = 30). **C** The Kaplan-Meier analysis of recurrence‑free survival of patients with different PLD3 expression levels (*N* = 2765). **D** The Kaplan–Meier analysis of disease‑free survival of patients with different PLD3 expression levels (*N* = 236). **E** Schematic illustration of p53/ZEB1-PLD3 feedback loop regulating breast cancer progression. *P < 0.05.

We determined PLD3, DNMT3B, and ZEB1 expression levels in 30 cases of primary breast cancer and the paired adjacent normal tissues, which results showed that PLD3 expression was lower in breast cancer tissues compared to normal breast tissue, while DNMT3B and ZEB1 expression were higher in breast cancer tissues (Fig. 7A). Furthermore, RT-qPCR revealed that miR-6796 expression was lower in breast cancer than in normal breast tissue (Fig. 7B). Clinically, we discovered that the PLD3_low_ breast cancer patients had a significantly poorer prognosis than the PLD3_high_ breast cancer patients (Fig. 7C, D). These findings indicate that PLD3 is a tumor suppressor gene and a possible independent prognostic factor for breast cancer.

## Discussion

In the present study, we aim to investigate the role of PLD3 in breast cancer progression. PLD3 overexpression inhibited CDK1 activity by binding to and phosphorylating it, resulting in mitotic arrest and consequently, inhibiting breast cancer proliferation. PLD3 and its embedded miR-6796 were co-expressed. DNMT3B, as a miR-6796 target gene, could bind to ZEB1, regulate DNA methylation of the PLD3 promoter, and influence PLD3 and miR-6796 expression. Furthermore, wild-type p53 can regulate PLD3 and miR-6796 expression by competing with ZEB1 for binding to the PLD3 promoter. Therefore, our results reveal a novel feedback loop between PLD3, miR-6796, p53wt, and ZEB1 in breast cancer progression. Moreover, we confirmed that PLD3 was decreased in clinical breast cancer tissues and correlated with the poor prognosis of breast cancer patient.

According to research, abnormal activation or expression of phospholipase is involved in tumor occurrence, development, proliferation, and metastasis. The PLD signaling pathway promotes tumorigenesis by interacting with other tumor regulators (Ras, PDGF, TGF, and kinases) [21]. Radiotherapy combined with PLD inhibitors (PLD1 and PLD2) significantly improved radiosensitivity in the MDA-MB-231 breast cancer cell line [22]. There have been few studies on PLD3 function, most of which have focused on Alzheimer’s disease [23], actin generation in myocytes [24], and the regenerative potential of hematopoietic stem cells [25]. The relationship between PLD3 and tumors was rarely investigated. We discovered that patients with high PLD3 expression had a higher disease-free survival rate than those with low PLD3 expression in the GOBO database (http://co.bmc.lu.se/gobo). Furthermore, PLD3 overexpression could inhibit breast cancer cell proliferation, whereas lower PLD3 expression could promote breast cancer cell proliferation in vivo and in vitro. IHC staining confirmed that the proliferation index Ki-67 decreased in PLD3-overexpressing breast cancer cells. Ki-67 is a nuclear protein associated with proliferating cells that can indicate cell proliferation activity. Its function is closely related to mitosis [26], implying that PLD3 may be involved in regulating the breast cancer cell cycle.

Multiple positive and negative regulatory factors regulate the cell cycle orderly and strictly. Accurate and strict regulation of the cell cycle by regulatory factors at all levels is the key to the normal progress of the cell cycle. Cyclins are programmed to be synthesized and degraded at different cell cycle stages to ensure orderly cell cycle progression. CDK is a group of serine/threonine protein kinases, whereas CKI is a small protein. Cell cycle progression depends on the activity of CDK complexes and corresponding cell cycle regulators [27]. Dysregulation of cell cycle control due to abnormal CDK activity is a common feature of most malignant tumors [28]. Cell cycle regulation involves three checkpoints, including G1/S, G2/M, and the mitotic spindle checkpoint. The loss of control of G1/S and G2/M checkpoints is the primary cause of the malignant proliferation of tumor cells. G2/M phase arrest is the most critical cell cycle protection barrier for DNA damage repair before cells enter mitosis, determining whether cells undergo mitosis or apoptosis [29]. Classical theory suggests that CDC2 and CDC25C play a crucial role in regulating mitotic entry and progression, and CDC2 activation and CDC25C expression increase could promote cells to enter mitosis [30]. Abnormal activation of the CDC2-CDC25C positive feedback regulation loop can cause cell cycle disorder, resulting in uncontrolled cell proliferation and tumor formation [31]. This study discovered that when PLD3 was overexpressed, the CDC2 phosphorylation increased, the CDC2 activity decreased, the CDC25C expression, and Cyclin B1 down-regulated. The tumor cells were arrested in the G2/M phase, making it difficult to enter mitosis. Further experimental verification demonstrated that PLD3 could bind to CDK, resulting in G2/M phase arrest, increased cell multinucleation, and promoted breast cancer cell apoptosis.

One of the most important tumor suppressor genes is p53 [32]. In normal cells, wild-type p53 can activate downstream target genes, such as GADD45, p21, Bax, Fas, Puma, and Cyclin, to regulate the cell cycle, repair DNA damage, induce cell apoptosis, and inhibit angiogenesis, thereby inhibiting cancer. When p53 is mutated, it loses its tumor suppressor function, inhibits wild-type p53 activity, which contributes to carcinogenesis [33]. p53 mutations are identified in 20–30% of breast cancers [34]. According to Linderholm et al. [35], mutant p53 expression levels were negatively correlated with patient survival and highly expressed in breast cancers that were highly metastatic and poorly differentiated. According to Vant et al. [36], mutant p53 can be used as a reliable indicator to assess the proliferative and invasive potential of breast cancer, as well as one of the indicators to assess breast cancer prognosis. We discovered two potential p53 binding sites on the PLD3 promoter through JASPAR analysis. PLD3 expression levels were high in p53 wild-type breast cancer cell lines and low in p53 mutant breast cancer cell lines, indicating that p53 may regulate PLD3 expression levels. Wild-type p53 can specifically bind to the PLD3 promoter region and significantly increase PLD3 expression, whereas mutant p53 does not affect PLD3. Therefore, we concluded that PLD3 is a wild-type p53 target gene and inhibits breast cancer proliferation by activating its transcription.

miRNA is a single-stranded small molecule non-coding RNA that does not encode proteins. It regulates gene expression at the post-transcriptional level via the degradation or silencing of mRNA [37]. miRNAs, upstream transcription factors, and downstream mRNAs are interconnected to form functional network modules that participate in various biological and pathological regulatory processes, including the phenotypic process of malignant tumors [38]. Numerous microRNAs related to breast cancer have been identified to regulate breast cancer cell proliferation, invasion, metastasis, and other biological behaviors [13, 14]. miRNAs are classified as intergenic or intronic based on their coding positions [15]. Intronic miRNAs that depend on the transcription of encoded genes are typically co-expressed with their host genes and function in concert with their host genes to carry out specific physiological or pathological functions. For example, miR-208 and its host gene MYH6 are involved in the occurrence of myocardial hypertrophy and fibrosis [16]. miR-33 and its host gene SREBF1 co-regulate cholesterol metabolism [17]. miR-126 assists its host gene EGFL7 in inhibiting prostate cancer invasion and metastasis [18]. However, the role of miR-6976 in tumor progression is still unclear. In our study, we revealed that miR-6796 functioned as a tumor suppressor gene in breast cancer proliferation when co-expressed with PLD3.

Genome-wide DNA methylation decreases, and CpG island methylation increases in tumor cells [39]. DNA methyltransferases (DNMTs) catalyze DNA methylation, but not all DNMTs family members perform this function. Only DNMT1 and DNMT3A/3B have methyltransferase activity; DNMT1 can maintain DNA methylation, while DNMT3A/3B can generate DNA methylation. The PLD3 promoter sequence revealed a CpG island, indicating that DNA methylation in the PLD3 promoter regulates PLD3 and its embedded miR-6796. Wild-type p53 also significantly increased miR-6796 expression through methylation, whereas mutant p53 did not affect miR-6796.

ZEB1 is a zinc finger structural transcription factor that belongs to the E-box-related protein family. It has zinc finger domains at the N and C terminals. ZEB1 is highly expressed in tumor tissues, including lung cancer [40], liver cancer [41], pancreatic cancer [42], colon cancer [43], and breast cancer [44], and is associated with tumor malignancy, particularly in breast cancer. Previous research has discovered that ZEB1 promotes breast cancer cell proliferation by down-regulating p21 and up-regulating CDK2 and CDK4 through the cell cycle [45]. Preliminary evidence suggests that epigenetic inheritance of the ZEB1 gene is related to breast cancer metastasis and recurrence [46]. Recently, Fu et al. discovered that the ZEB1/p53 axis plays an important role in breast cancer, where the N-terminal zinc finger domain of ZEB1 can bind to p53, catalyze p53 deacetylation, and promote its degradation. The p53 is less likely to be ubiquitinated, has a longer half-life, and is more stable when ZEB1 is silenced [39]. Besides, previous studies also demonstrated that P53 could transactive miR-200 family members and then repressed ZEB1/2 [47, 48]. A ZEB1 binding site on the PLD3 promoter was discovered after analyzing the PLD3 promoter sequence. We hypothesized that ZEB1 could regulate PLD3 promoter DNA methylation. Furthermore, we demonstrated that, in P53mut status, ZEB1 could bind to the PLD3 promoter region, recruit DNMT3B to the PLD3 promoter DNA region, and result in DNA methylation, thereby reducing PLD3 and miR-6796 expression. Simultaneously, ZEB1 can inhibit PLD3 and miR-6796 by down-regulating p53. Further research revealed that miR-6796 had two potential binding sites on the DNMT3B 3’-UTR region, and when the two binding sites were mutated, miR-6796 lost its regulatory effect on DNMT3B 3’-UTR activity. The above findings confirmed that DNMT3B is a target gene of miR-6796 and can negatively regulate DNMT3B.

In conclusion, we unveiled that a feedback loop of p53/ZEB1-PLD3 was involved in breast cancer progression (Fig. 7E). We proposed that PLD3 and miR-6796 can serve as prognostic factors and promising targets for breast cancer therapeutics. Nevertheless, a large sample of clinical data is required to determine the clinical applications of PLD3 and miR-6796.

## Materials and methods

### Cell culture and clinical specimens

The four breast cancer cell lines MCF7, MDA-MB-231, CAL51(p53wt), CAL51(p53KO), one normal breast cell line MCF10A, and 293FT cell line, were obtained from the Cell Bank of the Chinese Academy of Sciences (Shanghai, China). The cells were cultured at 37 °C in 5% CO_2,_ as previously described.

Breast cancer tissue samples were obtained from Tianjin Medical University Cancer Institute and Hospital (TMUCIH). Thirty cases of breast cancer tissue and the paired normal tissue were included in this study. All tumor samples were obtained from newly diagnosed breast cancer patients who had not received therapy before sample collection. This study was approved by the Institutional Review Board of TMUCIH. The written informed consent was signed by all participants.

### Plasmids, miRNAs, and siRNAs

The miR-6796 mimics/inhibitor, siRNAs targeting PLD3, p53, Zinc finger E-box binding homeobox 1 (ZEB1), and corresponding controls were purchased from RiboBio (China). The sequences that contain predicted binding sites or corresponding mutants were synthesized and inserted into the pGL3-basic vector (Promega, Madison, WI, USA) and co-transfected with pRL-TK vector (Promega) into cells for the luciferase reporter assay. The mutant constructs were created using a site-directed mutagenesis kit (TransGen, China). Furthermore, human PLD3, ZEB1, p53mut, and p53wt cDNAs were synthesized and inserted into the pcDNA3-HA vector. All specific sequences were listed in Table S1.

### Reverse transcription‑quantitative polymerase chain reaction (RT-qPCR)

Total RNA was isolated from tissues or cells using the *mir*Vana^TM^ miRNA Isolation Kit (Life Technologies, Grand Island, NY, USA) or TRIzol Reagent (Life Technologies) according to the standard protocol. For miRNA, reverse transcriptions were performed using the TaqMan^TM^ MicroRNA Reverse Transcription Kit (Life Technologies). The cDNA amplification was performed using the TaqMan^TM^ MicroRNA Assay Kit (Life Technologies) according to the manufacturer’s instructions. The mRNA expression was determined using the GoTaq qPCR Master Mix (Promega, Madison, WI, USA). GAPDH and U6 snRNA were used as the endogenous control. Gene expression fold changes were assessed using the 2^-ΔCt^ method. The median value was chosen as the cut-off value to categorize the samples as high and low PLD3 and miR-6796 expression. All primer sequences were listed in Table S2.

### Transfection and generation of stable expressed cell lines

According to the instructions, all transient transfections were performed using FuGENE HD Transfection Reagent (Promega). Cells were transfected with specific lentiviruses (RiboBio) for 48 h to construct stable cell lines following instructions. Puromycin (2 mg/mL) was used to select the infected cells for one week.

### 3-(4,5-dimethylthiazol-2-yl)-2,5-diphenyltetrazolium bromide (MTT) and colony-formation assays

For the MTT assay, cells were seeded in 96-well plates (2 × 10^3^ per well) after transfection for 48 h. Each well was added with 10 μL 5 mg/mL MTT for 4 h. The medium was discarded, and the violet crystals were dissolved in 150 μL DMSO. The absorbance was measured at 570 nm using a micro-plate reader (Bio-Rad, Richmond, CA, USA). The cell viability was measured at the indicated times.

For the colony-formation assay, 500 cells were seeded per well in each 6-well plate. After three weeks of incubation at 37 °C, the colonies were fixed with 4% paraformaldehyde (PFA) for 30 min at room temperature and stained with 2% crystal violet solution for 15 min, washed with PBS, and dried thoroughly. Then, colonies were counted under a microscope.

### Immunofluorescence (IF)

The cells were seeded into 24-well plates with glass coverslips and cultured overnight at 37 °C with 5% CO_2_. The next day, cells were washed with PBS three times before being fixed in a 4% formaldehyde for 30 min. Then, the cells were permeabilized in ice-cold PBS containing 0.5% Triton X-100 for 15 min. Following that, cells were blocked with 3% BSA for 1 h before incubating with primary antibodies for 3 h. Then, cells were incubated with tetramethylrhodamine-isothiocyanate (TRITC)- or fluorescein isothiocyanate (FITC)-conjugated secondary antibodies for 1 h at room temperature. DAPI was used to determine nuclear stains. Finally, coverslips were observed and imaged using a Zeiss fluorescence microscope.

### Chromatin immunoprecipitation quantitative polymerase chain reaction (ChIP-qPCR) analysis

ChIP-qPCR analysis was performed by the manufacturer’s recommendations (Millipore, Bedford, MA, USA) or with an isotype control as previously described [49]. The immunoprecipitated DNA was analyzed using qPCR with primers specific to the region, and the results were expressed as a percentage of the input. All primer sequences were listed in Table S3.

### Dual-luciferase reporter assay

293FT cells or CAL51 (p53KO) cells (5 × 10^4^ per well) were seeded in 12-well plates. The cells were transfected with 200 ng of the indicated firefly luciferase reporter plasmid, 200 ng of the expression plasmid, 50 nM siRNAs or miRNAs, and 20 ng of Renilla reporter using FuGENE HD for 48 h. pRL-TK Renilla reporter was used as a normalization control. The luciferase activities were determined by Dual-Luciferase Reporter Assay Kit (Transgene) according to the instructions.

### Western blot

The cells were lysed with RIPA buffer containing 1 mM PMSF (Solarbio, Beijing, China). BCA Protein Assay Kit (Thermo Fisher Scientific) was used to determine protein concentration. Protein samples were boiled, separated on 8–10% SDS-PAGE, and then transferred to polyvinylidene fluoride (PVDF) membranes (Millipore). The membranes were blocked for 1 h with 5% (w/v) skimmed milk at room temperature and incubated with primary antibody at 4 °C overnight. After washing with Tris Buffered Saline with 0.05% Tween (TBST) three times, the membranes were incubated with horseradish peroxidase (HRP)-conjugated secondary antibody for 1 h at room temperature. Enhanced chemiluminescence (ECL) reagent (Millipore) was used to visualize the blots. All antibodies were listed in Table S4.

### Immunoprecipitation (IP)

The cells were lysed with RIPA buffer containing 1 mM PMSF. The primary antibody was incubated with 1 mg of the cell lysate overnight at 4 °C. Protein A/protein G agarose beads (Santa Cruz, CA, USA) were used to collect immunoprecipitated proteins, then washed and resuspended in immunoprecipitation buffer (50 mmol/L Tris-Cl [pH 7.9], 50 mM NaCl, 0.1 mM EDTA). The boiled samples were detected by western blot.

### Immunohistochemistry (IHC)

The mice breast cancer tissues or tumors were fixed with formalin and embedded in paraffin. A rotatory microtome was used to cut the thick consecutive sections (4 μm). After deparaffinization, hydration, antigen retrieval, and endogenous peroxidase blocking, each slide was incubated with a specific primary antibody overnight at 4 °C and then incubated with HRP-conjugated secondary antibody for 1 h at room temperature. Diaminobenzidine was added for coloration. The slides were counterstained with hematoxylin and visualized under an inverted microscope imaging system (Olympus). PLD3, Ki-67, DNMT3B, and ZEB1 expressions were evaluated blindly by two pathologists. The staining intensity of positive tumor cells was scored with four scales: 0 (no staining), 1 (weak staining), 2 (moderate staining), and 3 (strong staining). The positively stained tumor cell percentage was scored as 0 (<10%), 1 (10–25%), 2 (26–50%), 3 (51–75%), or 4 (>75%). The total immunostaining score, ranging from 0 to 12, was the multiplier of staining intensity and positive percentage. The high expression was defined as a final staining score of R4.

### Flow cytometric analysis

For cell cycle distribution, cells were digested by 0.25% trypsin and added to 95% ethanol at 4 °C overnight. After centrifugation, the cells were stained with 500 μL of propidium iodide (PI; BD Biosciences) and incubated in the dark for 15 min. The cell apoptosis assay was carried out according to the instructions of the APC Annexin V Apoptosis Detection Kit (BD Pharmingen). The cells were digested and stained with 5 μL APC annexin V and 5 μL PI in 1 × binding buffer for 15 min at room temperature in the dark. All samples were analyzed using CellQuest software on a BD FACS Aria flow cytometer, and data were analyzed using FlowJo software.

### Bisulfite sequencing PCR (BSP)

The DNA were isolated from cells by genomic DNA isolation kit (Thermo, USA), and the DNA conversion was performed by a EpiTect bisulfite kit (Qiagen, USA). The oligonucleotides of PLD3 promoter region were amplified by polymerase chain reaction and retrieved by a EasyPure Quick Gel Extraction Kit (Transgene, China). Then the fragments were inserted to a pGEM-T easy vector system (Promega, USA). For each ligation, ten colonies were selected randomly, and sequenced by RiboBio (China).

### Xenograft

Female NOD/SCID/IL2 receptor g null (NSG) mice (4–6 weeks) were inoculated subcutaneously with 1 × 10^7^ cells. Each group included 6 mice and was randomly assigned without blinding. The tumor size was measured using an electronic vernier caliper every five days. Tumor volume was calculated using the following formula: tumor volume = (width × length)^2^/2. All the animals were executed after 35 days. The terminal volume and weight of tumor tissues were measured. All animal experiments were approved by the Animal Ethics Committee of TMUCIH and were performed according to the animal welfare guidelines in cancer research.

### The Kaplan–Meier plotter

The prognostic value of PLD3 expression was examined using the online database Kaplan–Meier Plotter (www.kmplot.com/mirpower). Patients were divided into two groups using the “auto select best cutoff” feature. The RFS and DFS of breast cancer patients were assessed using a Kaplan–Meier survival.

### Statistical analysis

SPSS 24.0 (IBM, Armonk) was used for data analysis. All experiments were independently repeated three times, and the measurement data were exhibited as mean ± standard deviation. A one-way ANOVA or Student’s *t*-test was used to determine group differences. *P* value < 0.05 was considered statistically significant.

### Supplementary information

Supporting information

Original Data File

## Data Availability

All data generated or analyzed during this study are included in this article and its supplementary information files.
