# Supplementary material for: The p53/ZEB1-PLD3 feedback loop regulates cell proliferation in breast cancer
Source: Cell Death Dis. 2023 Nov 17;14(11):751. doi: 10.1038/s41419-023-06271-4 (PMC10656518; doi:10.1038/s41419-023-06271-4)
Supplement: Supplementary file 1 — Supporting information [file 41419_2023_6271_MOESM1_ESM.docx]

**
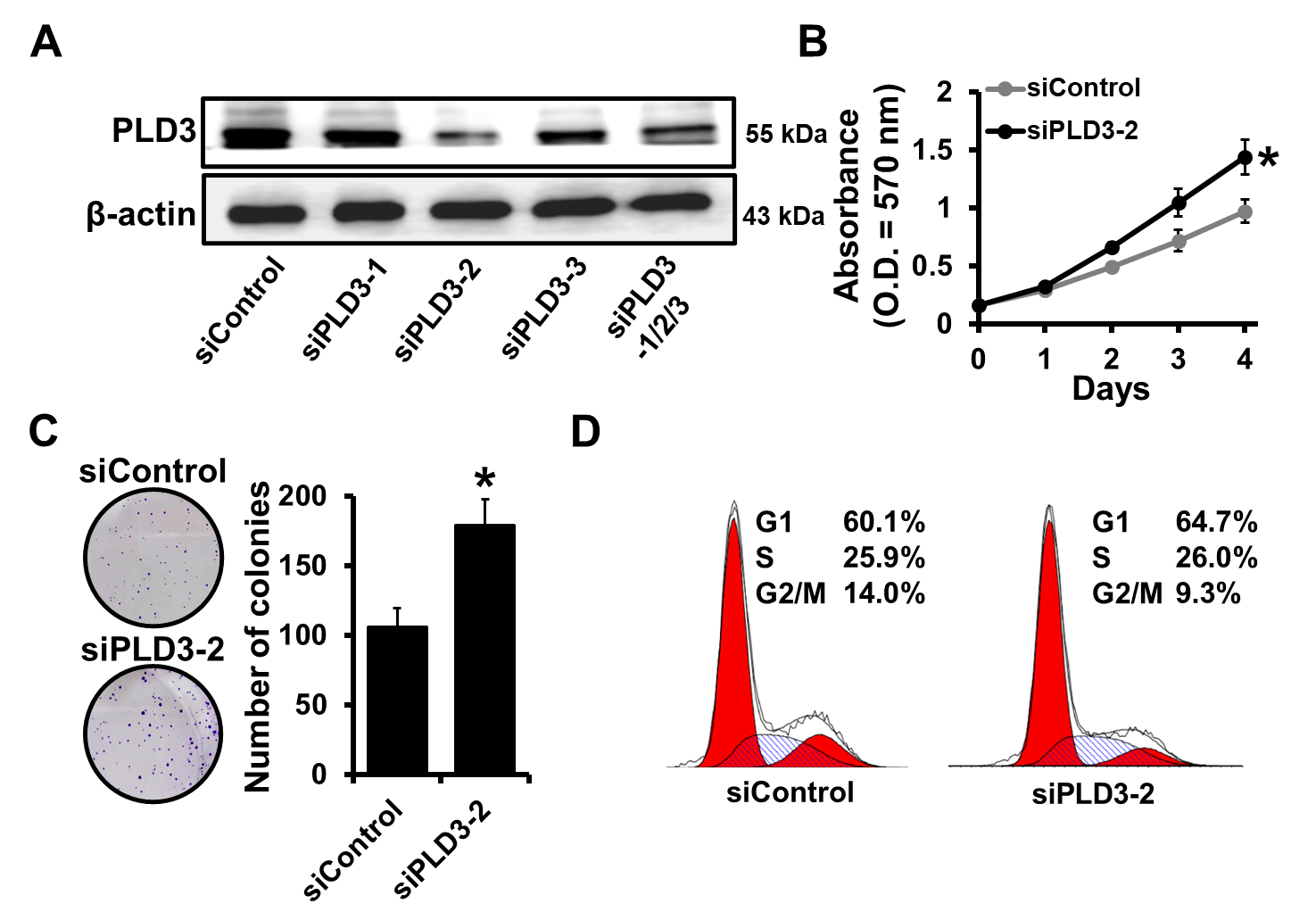
**

**Figure S1. PLD3 deletion promotes breast cancer cell proliferation. (A)** PLD3 expression in stable PLD3‑depleted cells (MCF7-shPLD3) and control cells (MCF7-shControl) was determined by western blot. **(B–C)** MTT (B) and colony-formation (C) analyzed the proliferation of the cells described in (A). **(D)** Flow cytometry analyzed the cell cycle distribution of the cells described in (A). **P* < 0.05.

**
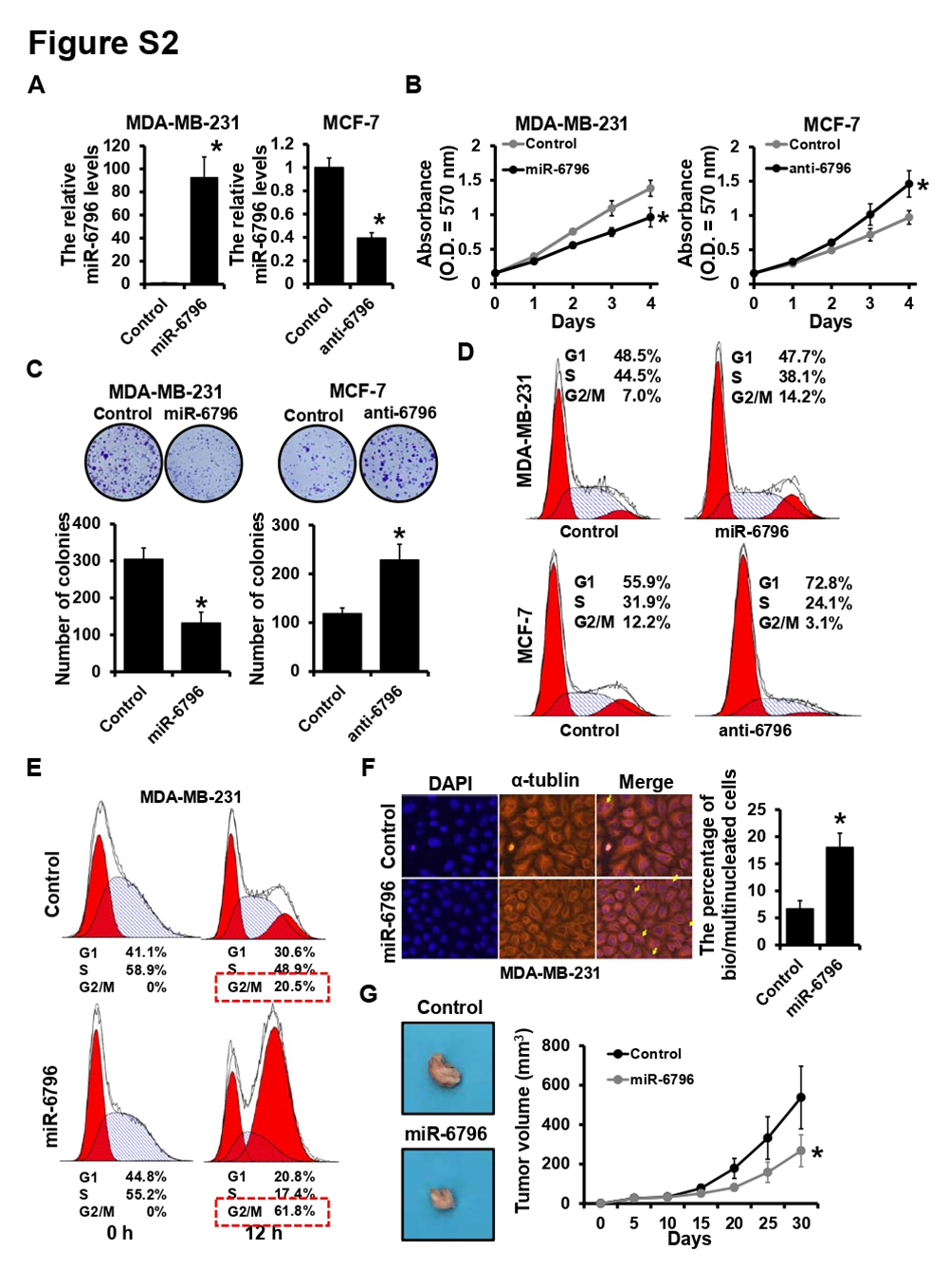
**

**Figure S2. miR-6796 inhibits the proliferation of breast cancer cells. (A)** The miR-6796 expression in MDA-MB-231 cells transfected with miR-6796 mimic and MCF-7 cells transfected with miR-6796 inhibitor, and control cells, were determined by RT-qPCR. **(B–C)** MTT (B) and colony-formation (C) analyzed the proliferation of the cells described in (A). **(D)** Flow cytometry analyzed the cell cycle distribution of the cells described in (A). **(E)** Double thymidine cell cycle synchronization method was used to synchronize breast cancer cells in G1/strand or pre-mitotic metaphase. The effect of miR-6796 expression levels on the cell cycle distribution of breast cancer was evaluated by flow cytometry. **(F)** The effect of miR-6796 on multinucleation in MDA-MB-231 cells was evaluated by IF. **(G)** Tumor growth curves of the subcutaneous tumor made of the miR-6796-overexpression and control cells at the indicated times and dissected tumors photographed at the time of harvest. **P* < 0.05.

**Table S1. Oligonucleotides of miRNAs and siRNAs**

| mimic control | UGUACCAAUUUCCAGUGGAGAU |
| --- | --- |
| inhibitor control | AUGGUGUUAUCAAGUGUAACAG |
| miR-6796 mimics | ThermoFisher 4464066 |
| miR-6796 inhibitor | ThermoFisher 4464084 |
| siControl | TTCTCCGAACGTGTCACGT |
| siZEB1 | GCTGTTGTTCTGCCAACAGTT |
| siPLD3-1 | AGCCTAAACTGATGTACCA |
| siPLD3-2 | CCCGGAGTTTCATCTACGT |
| siPLD3-3 | CCAATGAGCTGCCCATGAA |
| sip53-1 | GTAGATTACCACTGGAGTC |
| sip53-2 | ACTACTGAGTGACAGTAGA |
| PLD3 promoter P1 up | GGTACC GGCTGTTCAGTTTCCATTGT |
| PLD3 promoter P2 up | GGTACC GCAGGAGAATCCCTTGAACC |
| PLD3 promoter P3 up | GGTACC CCGGTGATACAGTCGGATTC |
| PLD3 promoter low | CTCGAG CAGATCGCACAGTACCTGCA |
| PLD3 up | CTGCGTATCCCCCAGCCTTGA |
| PLD3 low | ACTGGATCGGGCCTCAGAGCA |
| ZEB1 up | GGCTTTATGAAAGTTACAAA |
| ZEB1 low | GGCTTCATTTGTCTTTTCTT |
| P53 up | GTCACTGCCATGGAGGAGCC |
| P53 low | GTCTGAGTCAGGCCCTTCTG |

**Table S2. Oligonucleotides used for RT-qPCR**

| **Name** | **Sequence (5’ to 3’)** |
| --- | --- |
| miR-6796-5p | ThermoFisher 4440886 |
| U6 | CTCGCTTCGGCAGCACA  AACGCTTCACGAATTTGCGT |
| PLD3 up  PLD3 low | TGGCAGGTATTATGAGACAGG  CAGGCAAACATGGTGGGATT |
| DNMT3B up  DNMT3B low | CCTGCTGAATTACTCACGCCCC  GTCTGTGTAGTGCACAGGAAAGCC |
| ZEB1 up  ZEB1 low | TCAAAAGGAAGTCAATGGACAA  GTGCAGGAGGGACCTCTTTA |
| GAPDH up  GAPDH low | CAAGGTCATCCATGACAACTTTG  GTCCACCACCCTGTTGCTGTAG |

**Table S3. Oligonucleotides used for ChIP, dual-luciferase analysis and methylation specific PCR**

| **Name** | **Sequence (5’ to 3’)** |
| --- | --- |
| p53 site 1 up  p53 site 1 low | GACCAGCCTGACCAACATGC  TTCTTGATAATGACTAGTGC |
| p53 site 2 up  p53 site 2 low | GGACTATCTGCTCTCATTGT  CTCAGTTTCCTCCCCTGTGG |
| ZEB1 site up  ZEB1 site low | GGGAGGAAACTGAGGCTCAA  CAGTTCAGCTTCCGGCGGTG |

**Table S4. Antibodies used for study**

| **Name** | **Source** | **Catalog** |
| --- | --- | --- |
| HA | Immunoway | YM3003 |
| PLD3 | Abnova | PAB20624 |
| β-Actin | Immunoway | YM3028 |
| DNMT1 (D63A6) XP® Rabbit mAb | Cell Signaling | 5032 |
| DNMT3A (D2H4B) Rabbit mAb | Cell Signaling | 32578 |
| DNMT3B (E8A8A) XP® Rabbit mAb | Cell Signaling | 57868 |
| Cyclin A2 (E6D1J) XP® Rabbit mAb | Cell Signaling | 67955 |
| Cyclin B1 (V152) Mouse mAb | Cell Signaling | 4135 |
| Cyclin D1 (E3P5S) XP® Rabbit mAb | Cell Signaling | 55506 |
| Cyclin E1 (D7T3U) Rabbit mAb | Cell Signaling | 20808 |
| cdc2 (POH1) Mouse mAb | Cell Signaling | 9116 |
| Phospho-cdc2 (Tyr15) (10A11) Rabbit mAb | Cell Signaling | 4539 |
| cdc25C (5H9) Rabbit mAb | Cell Signaling | 4688 |
| p21 Waf1/Cip1 (12D1) Rabbit mAb | Cell Signaling | 2947 |
| p53 (1C12) Mouse mAb | Cell Signaling | 2524 |
| Anti-ZEB1 antibody | Abcam | ab180905 |
