# Supplementary material for: The p53/ZEB1-PLD3 feedback loop regulates cell proliferation in breast cancer
Source: Cell Death Dis. 2023 Nov 17;14(11):751. doi: 10.1038/s41419-023-06271-4 (PMC10656518; doi:10.1038/s41419-023-06271-4)

Figure 1

PLD3

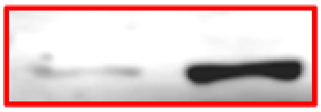

β-actin

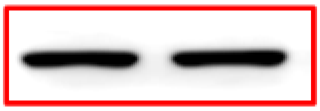

Figure 2

Cyclin B1

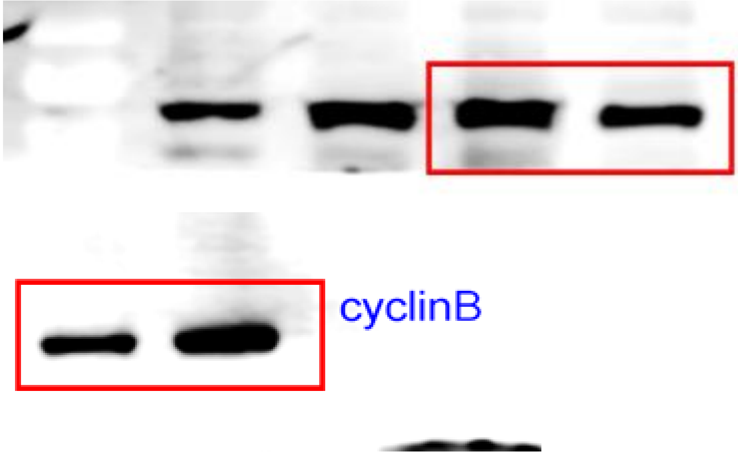

CDK1

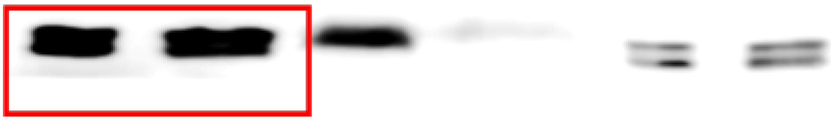

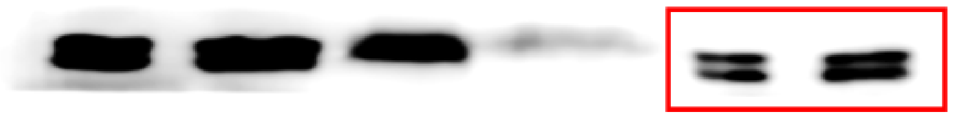

pCDK1

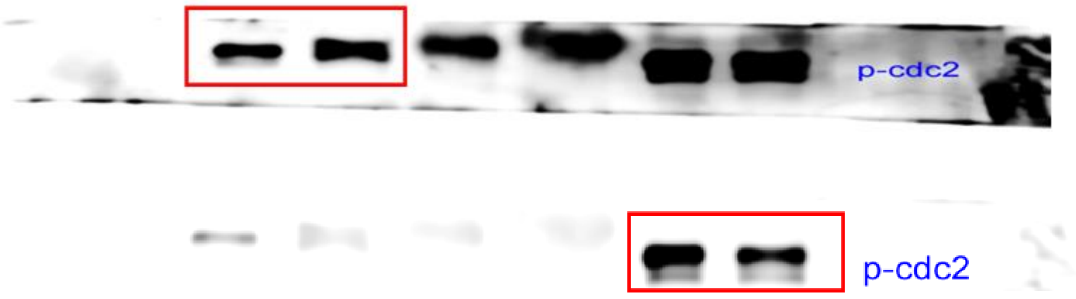

CDC25C

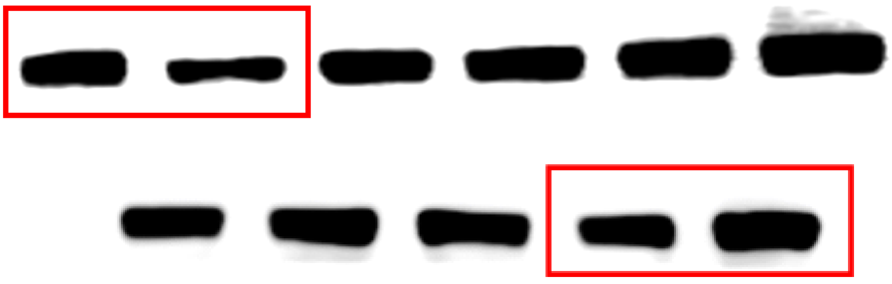

Cyclin E

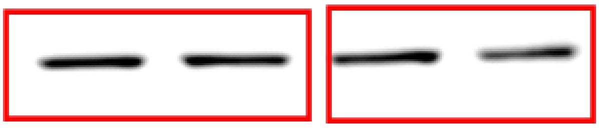

P21

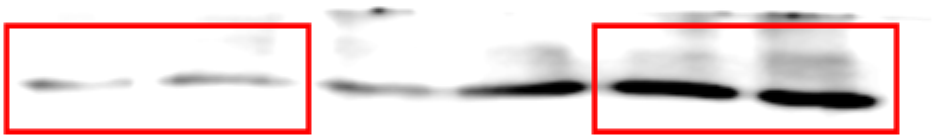

Cyclin A

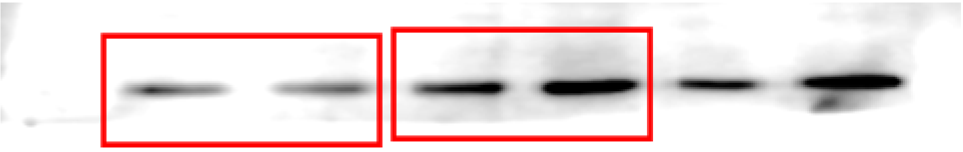

Cyclin D1

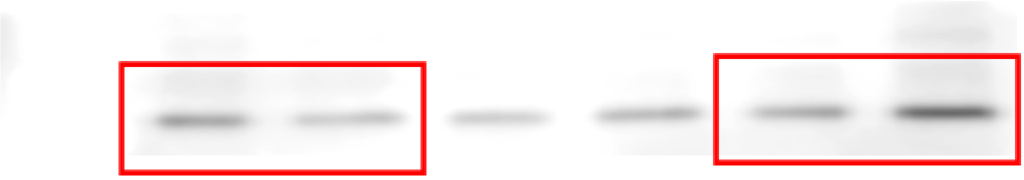

β-actin

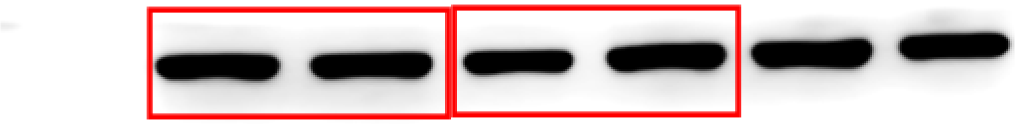

PLD3

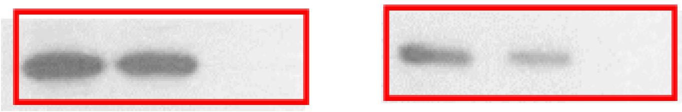

CDK1

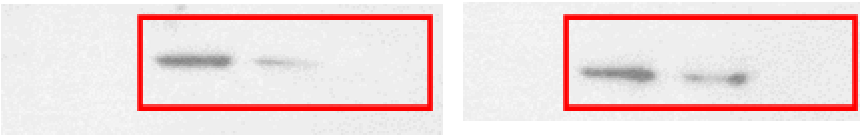

pCDK1

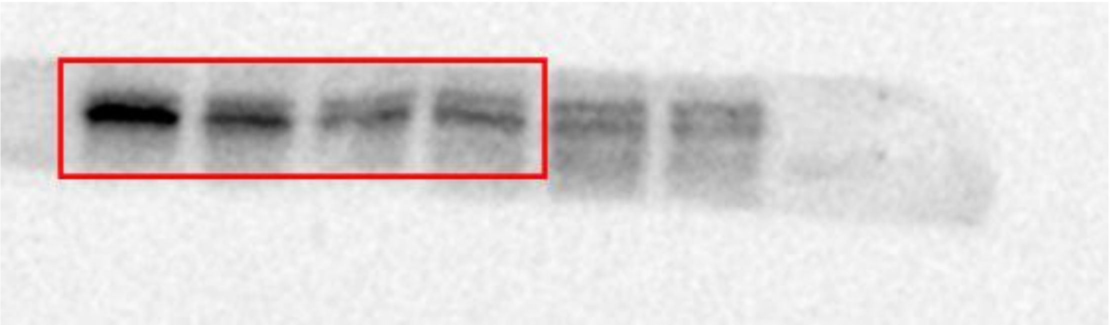

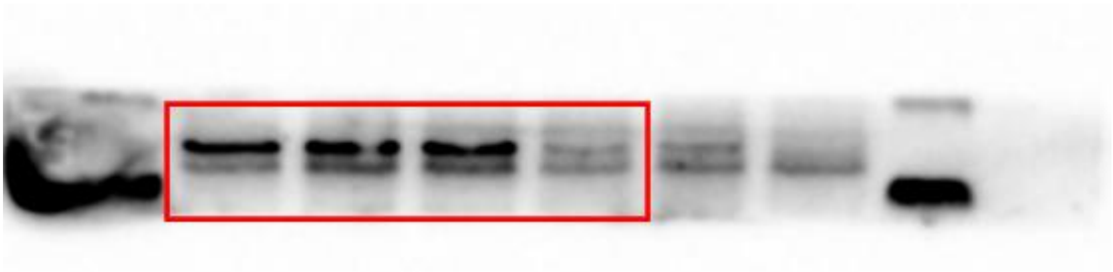

β-actin

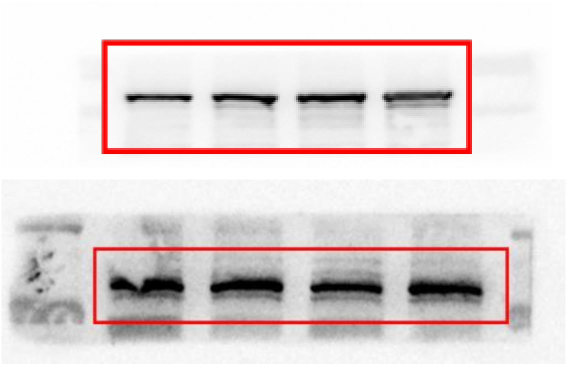

Figure 3

P53

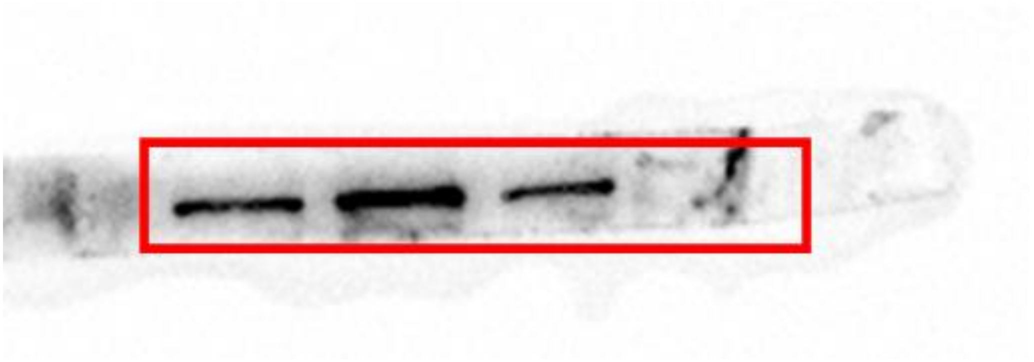

PLD3

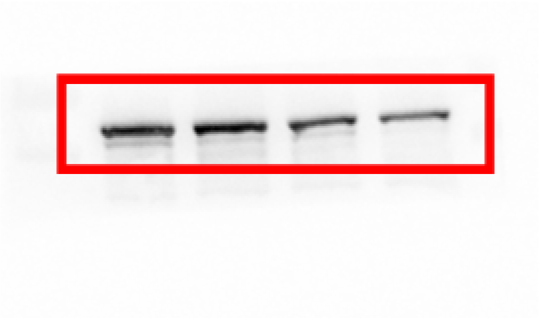

β-actin

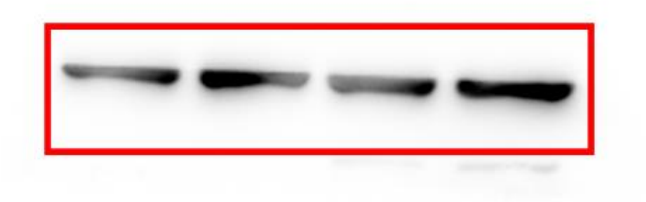

P53

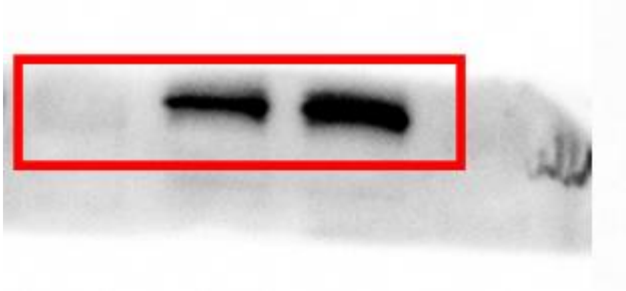

PLD3

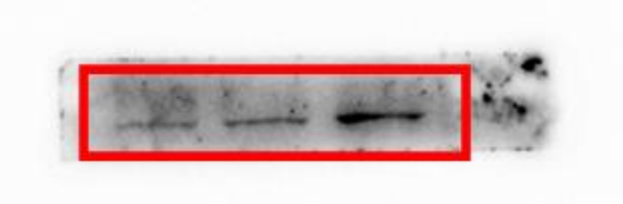

β-actin

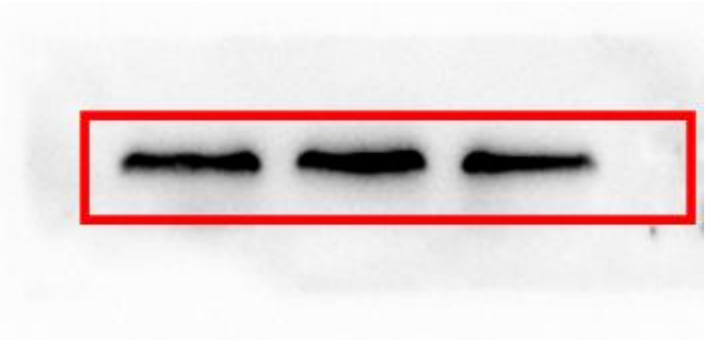

P53

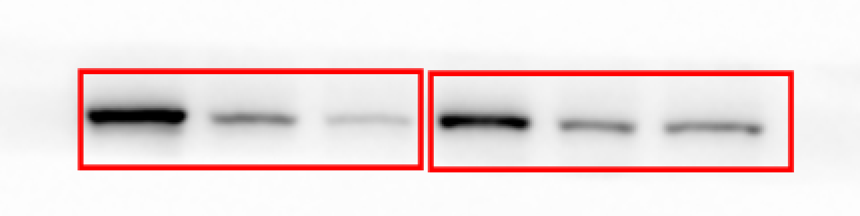

PLD3

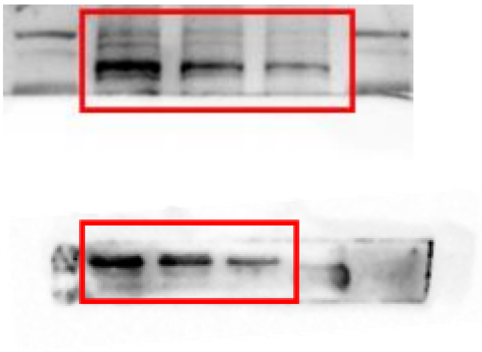

β-actin

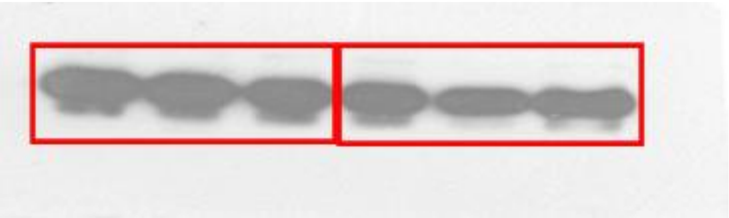

Figure 4

PLD3

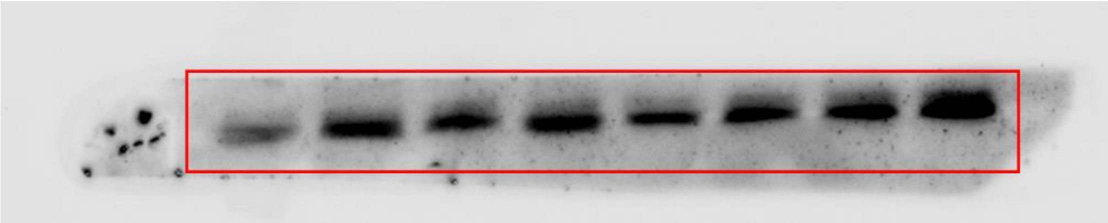

β-actin

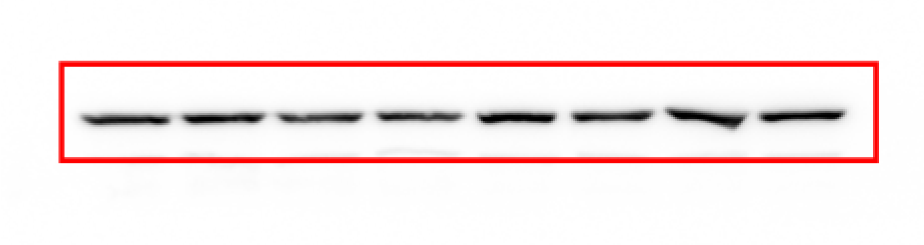

Figure 5

ZEB1

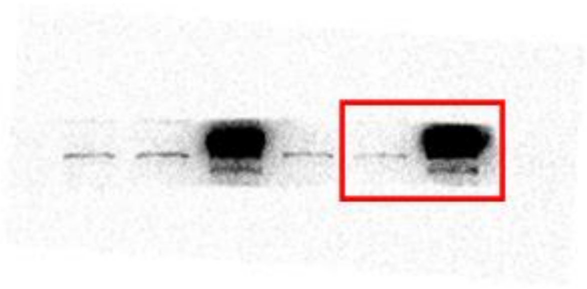

P53

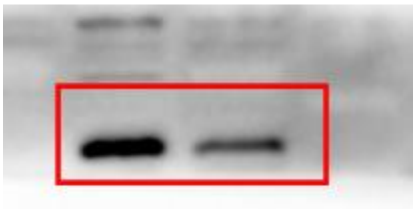

PLD3

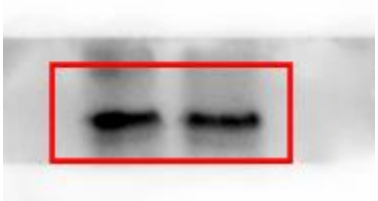

β-actin

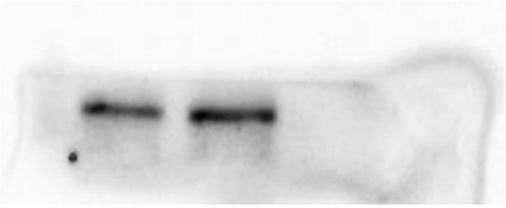

ZEB1

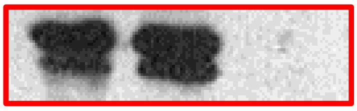

DNMT3B

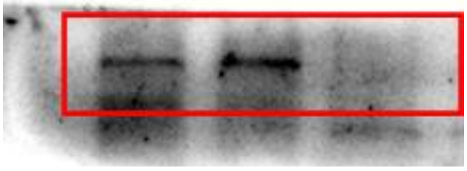

P53

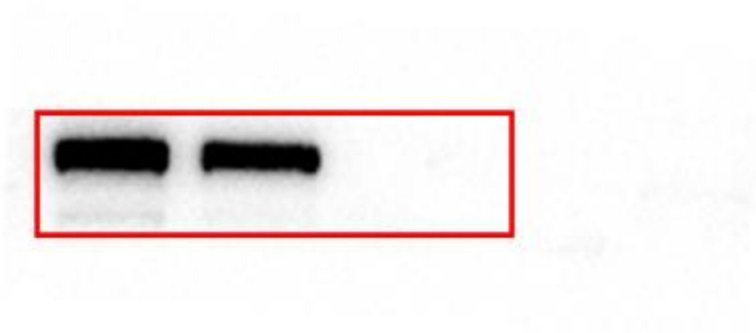

Figure 6

DNMT3B

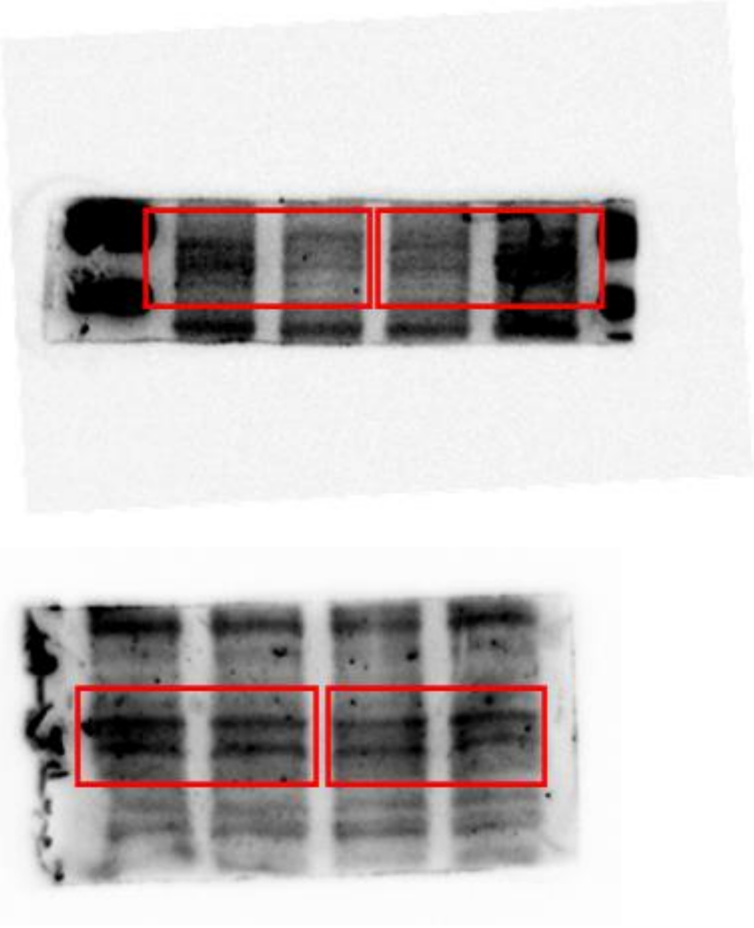

PLD3

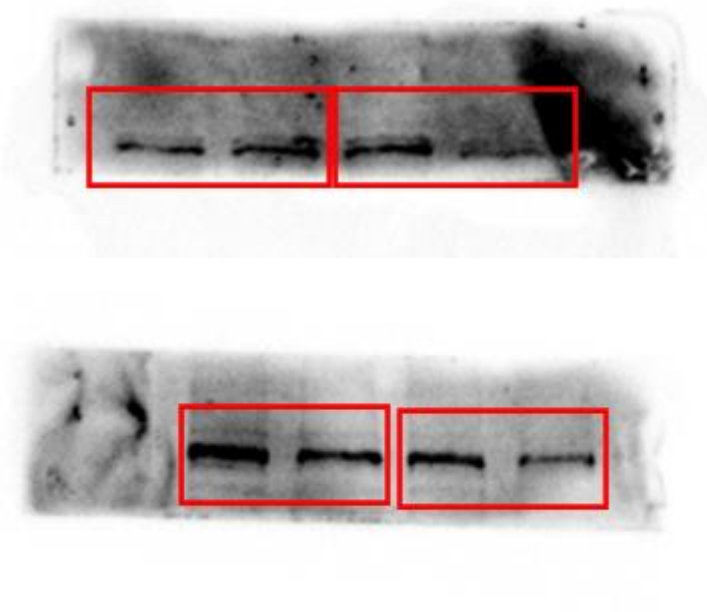

β-actin

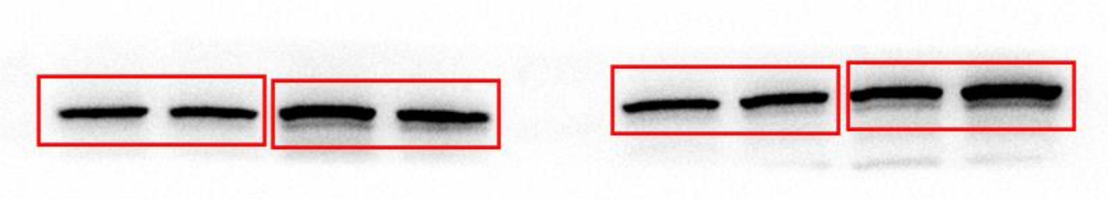

Figure S1

PLD3

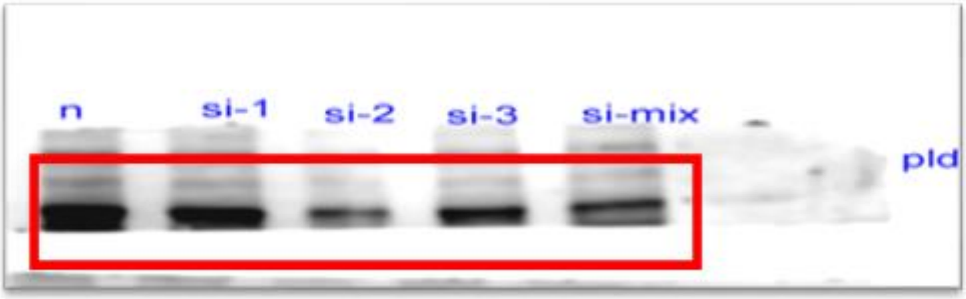

β-actin

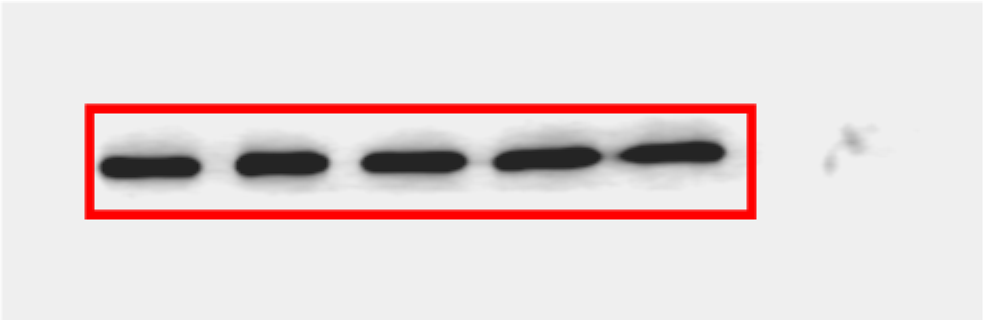

Supplement: Supplementary file 2 — Original Data File [file 41419_2023_6271_MOESM2_ESM.pdf]
